# Hinge-shift mechanism as a protein design principle for the evolution of β-lactamases from substrate promiscuity to specificity

**DOI:** 10.1038/s41467-021-22089-0

**Published:** 2021-03-25

**Authors:** Tushar Modi, Valeria A. Risso, Sergio Martinez-Rodriguez, Jose A. Gavira, Mubark D. Mebrat, Wade D. Van Horn, Jose M. Sanchez-Ruiz, S. Banu Ozkan

**Affiliations:** 1 Department of Physics and Center for Biological Physics, Arizona State University, Tempe, AZ USA; 2 Departamento de Quimica Fisica, Facultad de Ciencias, Universidad de Granada, Granada, Spain; 3 Unidad de Excelencia de Quimica Aplicada a Biomedicina y Medioambiente (UEQ), Universidad de Granada, Granada, Spain; 4 Laboratorio de Estudios Cristalograficos, Instituto Andaluz de Ciencias de la Tierra, CSIC, Universidad de Granada, Granada, Armilla Spain; 5 The Biodesign Institute Virginia G. Piper Center for Personalized Diagnostics, Arizona State University, Tempe, AZ USA; 6 School of Molecular Sciences, Arizona State University, Tempe, AZ USA; 7 Present Address: Departamento de Bioquimica, Biologia Molecular III e Inmunologia, Universidad de Granada, Granada, Spain

**Keywords:** Computational biophysics, Protein design, Biological physics

## Abstract

TEM-1 β-lactamase degrades β-lactam antibiotics with a strong preference for penicillins. Sequence reconstruction studies indicate that it evolved from ancestral enzymes that degraded a variety of β-lactam antibiotics with moderate efficiency. This generalist to specialist conversion involved more than 100 mutational changes, but conserved fold and catalytic residues, suggesting a role for dynamics in enzyme evolution. Here, we develop a conformational dynamics computational approach to rationally mold a protein flexibility profile on the basis of a hinge-shift mechanism. By deliberately weighting and altering the conformational dynamics of a putative Precambrian β-lactamase, we engineer enzyme specificity that mimics the modern TEM-1 β-lactamase with only 21 amino acid replacements. Our conformational dynamics design thus re-enacts the evolutionary process and provides a rational allosteric approach for manipulating function while conserving the enzyme active site.

## Abstract (web summary)

TEM-1 β-lactamase evolved from ancestral enzymes that degraded a variety of β-lactam antibiotics with moderate efficiency and degrades β-lactam antibiotics with a strong preference for penicillins. Here authors developed a computational approach to rationally mold a protein flexibility profile on the basis of a hinge-shift mechanism and show a putative Precambrian β-lactamase that mimics the modern TEM-1 β-lactamase with only 21 amino acid replacements.

## Introduction

Proteins are biomolecular machines with the capacity to participate in a wide variety of functions with remarkable efficiencies and specificities. Apart from being the efficient worker bees of the cell, proteins evolve and develop new functions over time; this process is critical for the evolution and survival of the organism. Proteins owe this remarkable capability to their three-dimensional (3D) network of atomic interactions, which orchestrates the communication between different parts of the protein chain in order to accomplish their designated functions. A complete understanding of the blueprint of their functional behavior (i.e., the relationship between their sequence, structure, dynamics, and function) and how it evolves with time could dramatically expand our ability to develop protein-based catalysts with potentially far-reaching applications to fields including chemistry, biotechnology, and medicine.

One such popular target of evolutionary studies is the TEM-1 β-lactamase. Modern β-lactamase enzymes are proteins that aid bacteria in their fight against antibiotics by hydrolyzing β-lactam antibiotics, like penicillin and cefotaxime (CTX), rendering the antibiotic useless. In an attempt to understand the molecular mechanism of antibiotic resistance in bacteria, particularly how variations at the sequence level impact function, these enzymes have been a target of a variety of evolutionary studies^1–10^. After its discovery in 1963^11^, ~170 other variants of TEM-1 β-lactamase have been isolated, making it one of the most understood and investigated enzymes from an evolutionary perspective^1,7–15^.

**Fig. 1:**
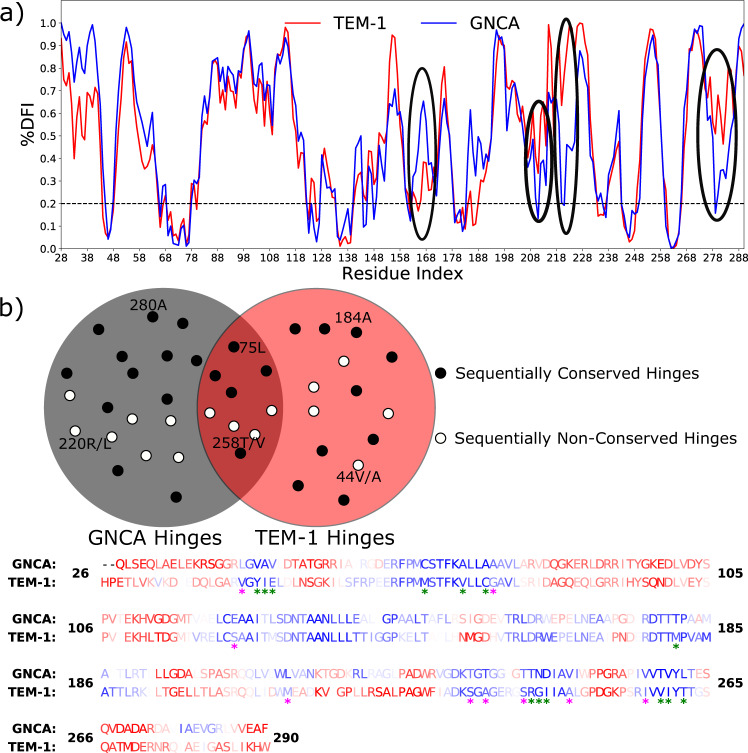
Differences in the flexibility profiles of GNCA and TEM-1 β-lactamases. **a** Comparison of the flexibility profile of ancestral β-lactamase (GNCA, blue) with the modern β-lactamase (TEM-1, red). We identify several regions focused around residues 166, 205, 223, and 280, where key differences (highlighted, black circles) in the dynamics of GNCA and TEM-1 β-lactamases, are observed. Typically, residues with the percentile rank of their DFI score (%DFI) <0.2 are deemed rigid hinges. These residues have been observed to play a critical role in the functional dynamics of the protein^28,55–58^. **b** A Venn diagram illustrating the amino acid conservation of select common and non-common hinges in GNCA and TEM-1 β-lactamase. The circles schematically (only a subset of hinges are graphed) represent residues in GNCA and TEM-1 β-lactamase with or without amino acid substitutions. The alignment of TEM-1 and GNCA β-lactamase amino acid sequences are color-coded based on their DFI scores. Blue is rigid and red is flexible, with a spectrum of blue–white–red based on their DFI values. We observe several residue positions with conserved rigidity through evolution (common hinges, 41 in total) in addition to shifts in hinges (non-common hinges, 10 in GNCA and 11 in TEM-1 β-lactamase). Many of these residue positions have conserved amino acid identity (sequentially conserved), while others have evolved into a different amino acid (hence, sequentially non-conserved). For example, residue 220 is arginine, which is a hinge position in GNCA, but has evolved to leucine in TEM-1 β-lactamase while losing its rigidity (shown as 220 R/L). The residue positions that have maintained their rigidity without conserving their amino acid identity are highlighted in the sequence alignment by a green asterisk. The residues where flexibility increased or decreased beyond 0.2 (non-common hinges) along with a substitution are highlighted with a pink asterisk. The computed data is provided as a Source Data file.

Through a Bayesian approach in a phylogenetic framework, the Precambrian nodes in the evolution of class-A β-lactamases have been resurrected^13^. This study provided us with sequences and structures of enterobacteria (ENCA), the last common ancestor of various Gram-negative bacteria (GNCA), and the last common ancestor of Gram-positive and Gram-negative bacteria (PNCA). Based on the estimates of divergence times, these enzymes existed about 1 Ga (ENCA), 1.5 Ga (GPBCA), 2 Ga (GNCA), and 3 Ga (PNCA). Comparison of ancestral β-lactamase enzymes with the extant TEM-1 β-lactamase revealed that these share several physical features—X-ray structures show that they share a common 3D fold (root mean square deviation, RMSD ~0.6 Å); pairwise sequence alignment between the sequences of GNCA and TEM-1 β-lactamases indicates ~50% conservation in their amino acid sequence (see Fig. 1b). In addition, they also shared the same composition and the shape of their catalytic active site^10,11^. Despite these striking similarities, GNCA and TEM-1 β-lactamases have very divergent catalytic activity. The resurrected Precambrian β-lactamases were found to degrade both penicillin and third-generation antibiotics (such as CTX) with moderate catalytic efficiency. Of course, third-generation antibiotics are a human invention and did not exist in the Precambrian. The results, however, support that Precambrian β-lactamases were moderately efficient promiscuous enzymes capable of degrading a variety of β-lactam substrates. However, with time, these have evolved into highly specific enzymes that selectively degrade penicillin with about two magnitudes of higher activity^10,13^. These results are in stark contrast to the common structure–function paradigm where the structure is thought to have a one-to-one correlation with function. Indeed, all ancestrally reconstructed proteins show that 3D fold is conserved as the function evolved through amino acid substitutions. This is in agreement with the CATH^16^ and SCOP^17^ databases showing that there is a limited number of 3D folds and proteins with strikingly low sequence similarity and divergent functions that can adopt a common fold^18^. Furthermore, a detailed analysis by Osadchy and Kolodny^19^ also shows that protein structures are generally more conserved than their sequence, thereby indicating that the structurally similar proteins can be very divergent in their sequences as well as their functions.

To date, all resurrected ancestral protein studies have shown that the evolution of a new function and/or adaptation to a new environment is always accomplished while preserving the 3D structure^12,18,20–26^. Our previous studies identify similar trends in other enzymes and proteins, including thioredoxins^27^, GFP^28^, and others^9,10,29^, which highlight the important role played by the conformational dynamics in enzyme evolution, where the structure–function model of the protein activity is replaced by an ensemble model^9,10,27–31^. In this model, the native state of a protein is represented by a collection of different conformations visited by the protein. The protein then samples these conformations through a broad range of motions from atomic fluctuations and side-chain rotations to collective domain movements. Therefore, in this model, the function of a protein is governed by the dynamics of sampling through this ensemble, as opposed to exclusively a dominant structure.

The ensemble model of protein dynamics also fits very well with protein evolution as it explains the emergence of a new function or modulation of a pre-existing function for adaptation to a new environment while conserving the dominant structure. Nature modulates function by performing a series of subtle modifications in the ensemble of the protein conformations such that the structure remains conserved, but the dynamics of the protein are now different by restricting the sampling of a group of conformers while allowing others. Indeed, studies on protein design through directed evolution have also highlighted the importance of conformational dynamics^32–37^. However, the underlying molecular mechanism for evolution, in particular, which position to substitute in order to modulate the protein conformational dynamics, presents a major challenge. This challenge also addresses the issue that the activities of rationally designed enzymes are almost always universally less efficient than their naturally occurring counterparts by a couple of orders of magnitude^38–40^. However, recent efforts in the designed enzyme methods have enabled the successful engineering of proteins with novel catalytic functions^25,35,39–41^. A rigorous comparison shows that natural and engineered enzymes have essentially equivalent substrate-binding affinity, yet drastic differences in catalytic rates. These low catalytic rates could only slightly be increased after rounds of directed evolution experiments that allow distal mutations^39^. Thus, a fundamental lack of knowledge about which mutations will modulate the conformational dynamics towards targeted functions currently prevents rational engineering of enzymes with native-like catalytic efficiency.

In our previous computational studies^9,10,27–29^, we have identified a common underlying hinge-shift mechanism that accounts for many functional features during protein evolution. Here, hinges are the regions in a protein with relatively lower flexibility, which link higher flexibility regions. These computational studies suggest that an enzyme evolves with subtle changes in dynamics, and concurrently its function, through a series of hinge-shift mutations by interchanging and altering distinct, flexible positions with rigid positions. In doing so, evolution exploits an allosteric network of interactions that can modulate the active site by making distal substitutions. Several other studies have also validated the critical role played by allosteric interactions for evolutionary trajectories of enzyme function^42–47^.

In an effort to further validate the hinge-shift mechanism, we have engineered the minimum number of necessary substitutions in the ancestral GNCA β-lactamase, such that the designed mutant is closer, in its activity, to that of the specialist extant TEM-1 β-lactamase. In order to do so, we use the dynamic flexibility index (DFI)^9,10,27–29,48–50^ and dynamic coupling index (DCI)^9,10,28,29,48,51–53^ as the metrics for quantifying the flexibility profile and the coupling profile of various residue positions in GNCA and TEM-1 β-lactamase. We propose a detailed mechanism, according to which selected substitution mutations are applied to GNCA, which gradually drives its dynamics (DFI profile) towards that of TEM-1 β-lactamase. This change in dynamics should cascade further to a change in the activity of the designed mutant. This is then tested and validated by functional assays, where we observed that, as predicted by our analysis, the activity of the designed mutant to degrade benzylpenicillin (BZ) increases 3-fold, whereas the activity for degrading CTX showed a remarkable decrease of 10,000-fold. These results indicate that through mutations predicted by the hinge-shift mechanism, we have rationally engineered the promiscuous GNCA β-lactamase into a specialist enzyme that mimics TEM-1. These results further highlight the significant role that allostery and conformational dynamics play in the functional evolution of enzymes.

## Results

### Evolution conserves the 3D structure of β-lactamase while changing the dynamics

To explain the functional differences between the ancestral and extant β-lactamases, we employ a comparatively recent and less explored dynamics–function paradigm, which helps relate protein function with dynamics. We use all-atomic molecular dynamics (MD) simulations (see “Methods”) to obtain the equilibrium dynamics of ancestral (GNCA) and extant (TEM-1) β-lactamases. Thereafter, we compute the residue-specific flexibility profiles of the two proteins using DFI (see “Methods”). Several computational studies have been performed that predict the relationship between the function and protein flexibility^27,48,49,51,54^. DFI analysis reveals regions of low flexibility and high flexibility in a protein. Furthermore, the residues belonging to low flexibility regions in a protein, called hinges, are critical in coordinating collective protein dynamics. These typically act as hubs for communication between different parts of the protein^28,55–58^. Various evolutionary studies on the flexibility profiles of proteins have revealed that residues with low flexibility have a higher propensity to be conserved in evolution, and mutations in such low flexibility regions usually prove to be detrimental for the function, particularly when it comes to pathogenic mutations^49,51,54,59^. On the other hand, residues found in the higher flexibility regions in a protein have a higher conformational/vibrational entropy and efficiently sample the conformational landscape. Therefore, these participate in functions demanding a higher mobility, including ligand recognition. Such regions are observed to be more prone to neutral or compensatory mutations throughout evolution and are more forgiving to the effects of amino acid substitutions.

Upon comparing the DFI profiles of the ancestral β-lactamase, GNCA with the extant TEM-1 β-lactamase, we identify various differences in their predicted flexibilities (Fig. 1a). This result is in agreement with our previous DFI analyses, where we provided insights into the puzzling question of how ancestral β-lactamases can degrade a variety of antibiotics, exhibiting promiscuity, unlike the specific modern homologs that can only inhibit penicillin, while maintaining the same structure^9,10,27,29^. The special structural dynamics associated with substrate promiscuity of ancestral β-lactamases was revealed by patterns of high DFI values in regions close to the active site, illuminating the flexibility required for the binding and catalysis of different ligands^10^. In the present study, we carefully further examined the changes in GNCA and TEM-1 β-lactamase DFI profiles by focusing on their respective DFI percentile ranking (%DFI). The %DFI gives the relative ranking of each residue, such that a residue with 0.1 %DFI score indicates that the residue is among the 10% least flexible residues. Comparing these, we observe a number of low flexibility residues in GNCA and TEM-1 β-lactamases (hinges exhibiting <0.2 %DFI), which retained their flexibility through evolution (also see Supplementary Fig. 1). We label these residues as common hinges. On the other hand, we also identified many hinge positions between GNCA and TEM-1, which underwent a significant change in their flexibility by increased dynamics or enhanced rigidity through evolution. Such residues are labeled as non-common hinges.

### Mimicking the dynamics of TEM-1 β-lactamase, by shifting the hinges in GNCA β-lactamase

In our previous analyses of other proteins including thioredoxins^27^, β-lactamase^9^, and GFP proteins^28^, we have shown that changes in the protein flexibility profile, through DFI, are able to accurately capture the changes in their function. Through computational studies, we have observed that during evolution, nature manipulates the dynamics of the protein by shifting its hinge residues—the hinge-shift mechanism, where some flexible residues become more rigid evolving into hinges, and alternatively, other rigid hinge positions become more flexible, leading to changes in dynamics to adapt to a new environment or to evolve a new function^9,27,29^. In order to perform these hinge shifts without sacrificing the 3D fold, the residues with moderate flexibility are usually substituted, making them rigid. This process is accompanied by a loss of rigid regions in the ancestral proteins in the form of compensation in order to preserve the protein fold and stability. Here, we attempt to manipulate ancestral β-lactamase (GNCA) dynamics such that it emulates modern β-lactamase (TEM-1) dynamics and function. To achieve this, we target a minimum number of substitutions at the positions involved in hinge shifts. This is done first in silico and then computational predictions are characterized experimentally in order to validate and gain a deeper understanding of the underlying mechanism of evolution for modulation of the function from exhibiting promiscuous activity towards antibiotics to becoming a specialist.

We first focus on the critical role played by hinges in GNCA and TEM-1 β-lactamases. With the help of the DFI flexibility profile, we have identified several hinge positions in the ancestral enzyme, GNCA, and the extant TEM-1 β-lactamase, which have preserved their low dynamic flexibility (common hinges), and also identified the positions that exhibit low flexibility either in GNCA or TEM-1 β-lactamase (non-common hinges), as shown in Fig. 1b.

**Fig. 2:**
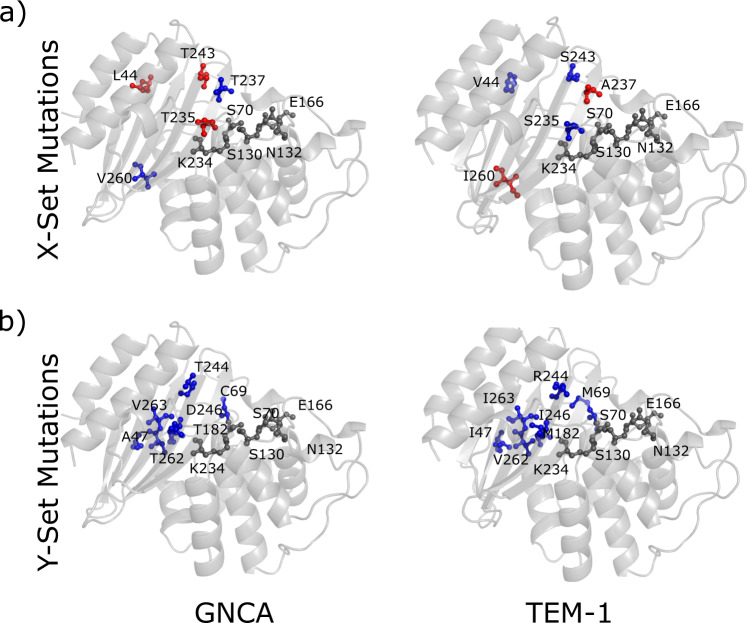
Residue positions selected for substitution in sets X and Y. **a** Non-common and sequentially non-conserved residues substituted in set X shown as sticks on the cartoon representation of GNCA and TEM-1 β-lactamase. **b** Common and sequentially non-conserved residues substituted in set Y shown as sticks on the cartoon representation of GNCA and TEM-1 β-lactamase. The substituting residues are colored based on their DFI profile where blue sticks represent residues with low DFI (hinge) and the red sticks represent residues with high DFI (flexible/non-hinge). The catalytic positions are shown in dark gray.

In order to shift the dynamics of GNCA towards TEM-1 β-lactamase, we aim to use a rational design principle of the hinge-shift mechanism where we attempt to reproduce the shift in hinge locations made through evolution. We, therefore, substitute the positions of GNCA residues with the corresponding amino acids identified in TEM-1 β-lactamase in the following two sets:

Non-common hinges and sequentially non-conserved residue positions (set X): here, we identify residue positions in GNCA β-lactamase, which have been substituted in TEM-1 β-lactamase while becoming either flexible (hinge-loss) or rigid (hinge-gain) (residues highlighted with a pink asterisk in Fig. 1b). As discussed earlier, such residue positions are expected to play an important role in describing the functional landscape of the protein. These substitutions typically lead to the modulation of dynamics and function (the hinge-shift mechanism for evolution^9,10,27,28^). Therefore, in this set, we follow this mechanism by considering sequentially non-conserved hinge residues in GNCA and TEM-1 β-lactamases for substitutions. In order to identify the minimum set of such hinges to replicate the desired change where it would shift the DFI profile of GNCA towards that of TEM-1 β-lactamase, we select only those non-common hinges in GNCA β-lactamase that are dynamically coupled with other non-common hinges in TEM-1 β-lactamase. The strength of coupling of a residue with another residue is quantified using pairwise DCI (see “Methods”), where we select only those residues that have a %DCI coupling score >~0.8 (see Supplementary Fig. 2a). %DCI represents the percentile ranking of the DCI score of residues. Therefore, a residue with a %DCI score >0.8 would imply that the residue is among the 20% of residues with the highest score. Further, in the DCI analysis, similar to the calculation of DFI, the coupling between different residues of GNCA and those of TEM-1 β-lactamase are calculated using covariance of fluctuations between pairs of residues obtained through MD simulations (see “Methods”). These selected GNCA β-lactamase residues are then substituted with the amino acids at corresponding residue identities of TEM-1 β-lactamase. These residues are shown in Fig. 2a.

Common hinges and sequentially non-conserved residue positions (set Y): as shown in Fig. 1, there are many residues with a conserved low flexibility, (i.e., remained as hinges) during the evolution from GNCA to TEM-1 β-lactamase (residues highlighted with a green asterisk in Fig. 1b). However, many of these positions are substituted in TEM-1 β-lactamase as we call them sequentially non-conserved common hinges. This suggests that these common hinges are crucial for the dynamics associated with the function. Therefore, these positions must exhibit long-distance communication with other non-common hinges within the 3D interaction network. Because of the shifts in hinges in other parts of the protein, some of these dynamically conserved positions (aka common hinges) need to be substituted to compensate the change in flexibility of the hinge-shift substitutions in other parts. We identify the sequentially non-conserved and common hinges in GNCA and TEM-1 β-lactamase, which exhibit high dynamic coupling to the other sequentially non-conserved and non-common hinge positions obtained using the pairwise DCI among them (Fig. 2b). Moreover, as seen in Supplementary Fig. 2b, residue 182 (which is also a non-conserved common hinge, exhibiting %DFI values of 0.07 and 0.18 GNCA and TEM-1 β-lactamase, respectively) is selected in mutation set Y despite being coupled to a lesser number of non-common and sequentially non-conserved hinges. Residue 182 was selected to ensure that common hinge positions that are equally distributed all over the protein sequence are selected, rather than biasing towards a specific region. Furthermore, residue 182 is the only non-conserved common hinge position that exhibits strong dynamic coupling with the key non-common and non-conserved hinges. Additional details for the selection of 182 and its dynamic couplings are provided in Supplementary Fig. 3. Moreover, as shown in Supplementary Fig. 6, excluding the 182 mutation from the Y set has a deleterious effect on the dynamics of GNCA β-lactamase. The impact on dynamics of exclusion of mutation at 182 makes intuitive sense, because residue 182 has been observed to play an important key role in several other evolutionary studies involving laboratory and clinical isolates^14,60^. Afterwards, we substitute the residue positions selected in mutation set Y in GNCA β-lactamase with the amino acids in the corresponding residue indices in TEM-1 β-lactamase (Fig. 2b).

We analyzed the flexibility profiles of the GNCA mutant models containing mutations from set X (GNCA-X), set Y (GNCA-Y), and also mutations from the combination of both (GNCA-XY). In order to do so, we initially ran long MD simulations of the mutants (≥400 ns or until they converge, see “Methods” and Supplementary Table 1 for details). Since our earlier work has demonstrated that DFI profiles correlate well with associated function^10,18,22–24^, the DFI profiles of X, Y, and XY mutants are computed from the equilibrated dynamics. We employ the use of principal component analysis (PCA) for clustering in order to evaluate the impact of these substitutions on the dynamics (the DFI profile) of GNCA β-lactamase. We align and arrange the DFI profiles of the mutants along with those of GNCA and TEM-1 β-lactamase such that each protein can be represented by a vector of dimension *N*, where *N* represents the total number of residues in each protein. Afterwards, we compare the projection of the vectors representing proteins along the lowest principal components in the vector space in order to observe the salient features differentiating them (see “Methods” for more details).

**Fig. 3:**
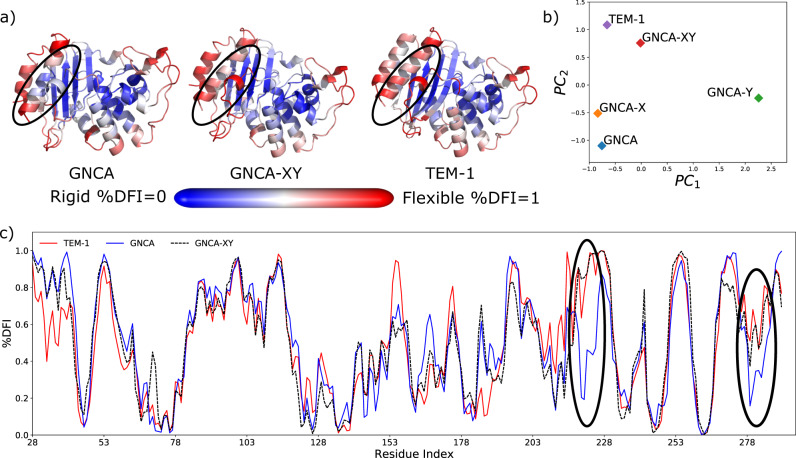
The similarities in the flexibility profiles of GNCA-XY mutants compared to the wild-type GNCA and TEM-1 β-lactamase. **a** Color-coded DFI profiles mapped onto the 3D structure where red is flexible and blue is rigid, **b** clustering of these profiles using principal component analysis (PCA), **c** the plot of mapped DFI profiles per residue position for each protein. The PCA analysis shows that GNCA-XY is similar to TEM-1 β-lactamase as shown in the principal component biplot. GNCA-XY mimics the flexibility profile of TEM-1 β-lactamase more closely than mutation sets X and Y alone, particularly around residues 220 and 280 (highlighted) in panel (**c**), also seen in their cartoon representations color-coded with the flexibility profile of their residues, red being flexible and blue rigid. The computed data are provided as a Source Data file.

First, we compare the lowest principal component of the flexibility profile of single set mutants GNCA-X and GNCA-Y with the wild type (Fig. 3b). We observe that set X has impacted the dynamics of GNCA such that a few of its residues share their dynamic flexibility with TEM-1 β-lactamases, creating hinge shifts particularly around residues 185 as shown by comparing their DFI profile (see Supplementary Fig. 4). Also, set Y significantly changes the GNCA flexibility profile, and as a result, GNCA-Y no longer shares the dynamical similarities with either of the two enzymes. While set X represents a fraction of the non-common and non-conserved hinges that exhibit dynamic coupling with the rest, mutating all of the non-common and non-conserved hinges also significantly alters dynamics of the catalytic pocket of GNCA β-lactamase (see Supplementary Fig. 5). This suggests that mutating all hinge-shift positions is not sufficient for driving the flexibility profile GNCA towards TEM-1 β-lactamase (see Supplementary Fig. 5). The common but substituted hinge sites (i.e., Y set substitutions) could play a vital role in compensating for these changes and contribute towards the desired flexibility profile of TEM-1 β-lactamase. Indeed, we observed that mutations in sets X and Y, when combined together, bring the dynamics of GNCA closer to TEM-1 β- lactamase (Fig. 3). Set XY mutations are able to induce hinge shifts in the GNCA β-lactamase around residues 220 and 280, which bring the XY mutant flexibility profile closer to TEM-1 β-lactamase (Fig. 3). This is also reflected by the two lowest principal components obtained from PCA comparing the DFI profiles of mutants GNCA-XY and GNCA-X with the wild type where we see that mutant GNCA-XY is comparatively closer to TEM-1 β-lactamase (Fig. 3b), suggesting that substitutions may make the designed ancestral enzyme more specific. In addition, this analysis also indicates that mutations from sets X and Y have a nonadditive impact on the dynamical landscape of GNCA β-lactamase. This points towards a possible role of epistasis^32,33,61–63^ between the mutation from sets X and Y.

Even though the clustering analysis shows similarities between the flexibility profiles of the designed mutant and wild-type TEM-1 β-lactamase, we still see that there are some significant differences between the hinges of TEM-1 β-lactamase and mutant GNCA-XY. Particularly around residues 203–215, 180–190, and the C terminus of the protein, the mutant was not able to replicate the flexibility profile of TEM-1 β-lactamase. Particularly, some of these positions where mutations failed to recapitulate flexibility profiles of TEM-1 β-lactamase correspond to the sequentially conserved non-common hinges; hence, we could not simply introduce substitutions by inferring the sequence variations between the ancestral and the extant β-lactamases as we did for the other non-conserved and non-common hinges. Therefore, in an attempt to bring the GNCA-XY mutant flexibility profile even closer to TEM-1 β-lactamase, we now focus on the flexible sites that exhibit allosteric dynamic coupling interactions with the catalytic site. These distal sites, which are highly coupled to the catalytic sites through an allosteric network of dynamic interactions, are called dynamic allosteric coupling (DARC) spots. These play a critical role in the evolution of protein dynamics towards new function^9,29,52,59^.

### Role of allostery through noninvasive DARC spots (set Z) brings mutant GNCA-XY closer to TEM-1 β-lactamase

Through our previous ancestral studies, we have observed that nature introduces substitutions at the DARC spots that are distal from the active site, where the mutations on DARC spots act as small perturbative changes, and distally modulate the dynamics of the protein/catalytic site in order to evolve a new function or adapt to new environment^9,27,29^. Indeed, this is also true for β-lactamases. First, we observed that a large fraction of the mutations (the clinically isolated mutations or those that emerged from directed evolution) are far from the active site, yet they modulate the equilibrium dynamics of the protein to confer resistance to antibiotics^9,27,29,53,59^. Furthermore, the DFI and DCI analysis of the exhaustive set of ~5000 mutations in TEM-1 β-lactamase^15^ have shown that the sites exhibiting mid-range flexibility and high dynamic coupling with the active site contribute most to the emergence of degrading different antibiotics^9,27,29^.

Therefore, in order to emulate nature, we also identify DARC spots, which are mid-flexible residues (having a DFI range of 0.3 < %DFI < 0.5) that are allosterically coupled to the active^9,29,52,59^. Since the active site positions are the rigid sites exhibiting low DFI throughout the evolution, they form a part of the sequentially conserved and common hinges. Moreover, by introducing substitution at DARC spots, we also aim to induce hinge shifts at non-common hinges, which are also sequentially conserved. Hence, we only select the DARC spot residues that are not only distal, >8 Å away from the active site, but also exhibit high dynamic coupling (%DCI > 0.8) with such non-common and sequentially conserved hinge sites (see Supplementary Fig. 7). These steps were taken in order to minimize the deleterious impact of the substitutions on the thermal stability and the kinetic activity of the protein. The residues closer to the active sites typically exhibit higher dynamic coupling as they are directly interacting. However, the substitutions at those sites are also more invasive and most likely impact the dynamics, and hence the function. Therefore, selecting DARC spots reduces the perturbative impact of mutations as they are relatively more flexible, which agrees with our earlier proteome-wide analysis showing that evolving sites are usually focused to flexible sites. Due to high degrees of freedom at those sites, they can compensate for changes upon substitution^29,51,59^. However, these DARC spots are not only flexible but also exhibit high dynamic coupling with the active or functionally important hinges sites. Therefore, the protein dynamics can tolerate substitutions at such residues, thereby allowing us to fine-tune the dynamics of the other functional sites^9,10,27–29,48–50^. Furthermore, the fact that these DARC spots exhibit high dynamic coupling with the non-common and sequentially conserved hinges allow us to exploit their compensatory network interactions to modulate the flexibility of these hinges.

**Fig. 4:**
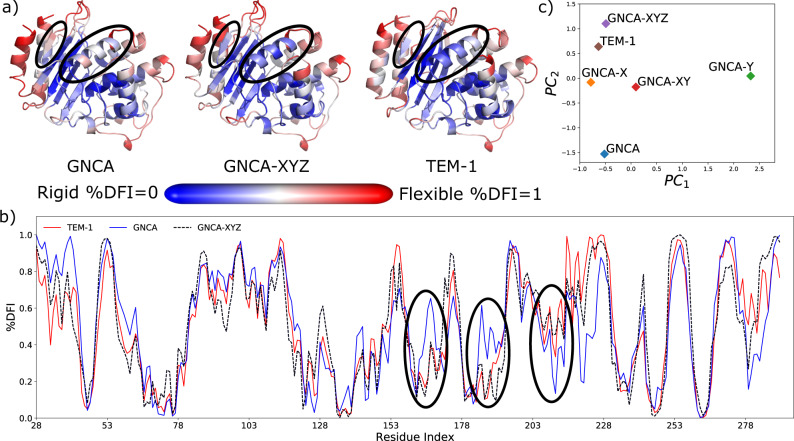
Substitutions from set Z bring the dynamics of GNCA-XY closer to TEM-1. Comparison of the flexibility profile of wild-type GNCA and TEM-1 β-lactamase with the GNCA mutants created by performing mutations from set Z over GNCA-XY (GNCA-XY) as **a** color-coded DFI profiles mapped onto the 3D structure where red-colored residues are flexible and blue are rigid, **b** clustering of these profiles using principal component analysis, **c** the plot of mapped DFI profiles per residue position for each protein. The PCA analysis presents that GNCA-XYZ is the closest to TEM-1 β-lactamase, when their difference is plotted in biplot using the first two principal eigenvectors. As observed per position DFI plot, GNCA-XYZ very closely mimics the flexibility profile of TEM-1 β-lactamase, particularly ~185, 155, 210, and the C terminus (highlighted regions in **b**). This is also seen in their cartoon representations (**a**). The computed data are provided as a Source Data file.

Thereafter, we performed MD simulations of the mutant (see “Methods”) generated by performing mutations from set Z on GNCA-XY mutant (GNCA-XYZ) and obtained its DFI profile from equilibrated dynamics. We then analyzed the impact of Z set on GNCA-XY by clustering its DFI profile with the DFI profiles of the wild-type GNCA and TEM-1 β-lactamase and those of other mutants, as shown in Fig. 4. The comparison of the flexibility profiles of the GNCA mutant with mutations from set XY and set Z together and the wild-type GNCA and TEM-1 β-lactamases (Fig. 4a, b) show that the mutant successfully recapitulates the dynamics of TEM-1 β-lactamase. It is able to capture the shortcomings of GNCA-XY mutant in regions close to 180–190, 203–215, and 153–157. This is also indicated by the clustering of the mutants from sets X, XY, and XYZ with the wild-type proteins by comparing their first two principal components, Fig. 4c. We observe that, as expected, the mutant GNCA-XYZ lies very close to the wild-type TEM-1 β-lactamase, which is an improvement over mutants with mutations from sets X and XY, suggesting that the ancestral variant GNCA-XYZ with merely 21 substitutions should degrade only penicillin with better efficiency than GNCA β-lactamase, mimicking the catalytic activity of TEM-1 β-lactamase. Therefore, using these 21 substitutions, we are able to dynamically replicate the effect of a total of 119 substitutions observed between GNCA and TEM-1 β-lactamase.

### Experimental characterization confirms antibiotic specificity of GNCA-XYZ

Based on the mutation sets discussed above, the mutants GNCA-X, GNCA-XY, and GNCA-XYZ were synthesized and their activities against the antibiotics CTX and BZ were characterized (see “Methods”). The experimental characterization of wild-type ancestral and extant β-lactamases shows that, as expected, GNCA β-lactamase is promiscuous and nonselective in its activity towards antibiotics BZ and CTX (with turnover rates of 0.3 and 1.2 s^−1^ μM^−1^, respectively). On the other hand, evolution has turned TEM-1 β-lactamase into a specialist, which preferentially catalyzes only BZ with a higher turnover rate (26 s^−1^ μM^−1^) in contrast to CTX (2.6 × 10^−3^ s^−1^ μM^−1^).

The GNCA-X mutant identified from our computational analysis reduced the turnover rates for CTX (2.5 × 10^−4^ s^−1^ μM^−1^). However, it has also diminished BZ activity by reducing its turnover rate by a factor of ten (0.03 s^−1^ μM^−1^). Likewise, substituting all the non-common and non-conserved hinges (GNCA-All_NN_) also yields a similar loss in function, suggesting that whether a fraction (set X) or all substitutions at non-common, non-conserved sites alone are not sufficient to engineer a penicillin-specific GNCA. We did not perform an experimental characterization of GNCA-Y as it was rejected in the initial round of computational analysis (see above). In addition, as discussed earlier, due to the compensatory network of interactions between X and Y set, the turnover rates of the mutant with the combined mutations from X and Y (GNCA-XY) showed some improvement over GNCA-X (as predicted to be slightly closer to TEM-1 β-lactamase) as its turnover the rate for BZ did not show appreciable change (0.22 s^−1^ μM^−1^). The mutant, however, showed a remarkable decrease in its turnover rate for CTX (2.6 × 10^−5^ s^−1^ μM^−1^), thus agreeing with the predicted analysis that introduced substitutions slowly drives GNCA β-lactamase from promiscuity to specificity, by reducing the catalysis of CTX while preserving the turnover rate for catalysis of BZ. Interestingly, with the addition of substitutions at DARC spots from set Z, the mutant GNCA-XYZ becomes more specific in its activity towards BZ by a three-fold increase in its turnover rate (0.9 s^−1^ μM^−1^). Moreover, its turnover rate towards CTX also showed a significant reduction (<1 × 10^−4^ s^−1^ μM^−1^) (see Supplementary Fig. 10), making it more preferential towards BZ, which is a functional characteristic of TEM-1 β-lactamase. These data are shown in more detail in Table 1.

**Table 1:** Experimental characterization of wild-type β-lactamase GNCA and TEM-1 β-lactamase, and the mutants created by mutation sets X, Y, and Z by calculating their turnover rates for catalysis of antibiotics benzylpenicillin (BZ) and cefotaxime (CTX).

Protein	BZ *k*_cat_/*K*_M_ (s^−1^ μM^−1^)	CTX *k*_cat_/*K*_M_ (s^−1^ μM^−1^)
TEM-1	26 ± 4.7	2.6 × 10^−3^ ± 1 × 10^−^^3^
GNCA	0.3 ± 0.1	1.2 ± 0.3
GNCA-X	0.03 ± 0.01	2.5 × 10^−^^4^ ± 5 × 10^−5^
GNCA-XY	0.22 ± 0.1	<1 × 10^−4^
GNCA-XYZ	0.9 ± 0.3	<1 × 10^−4^
GNCA^T235S_T237A_T243S^	0.03 ± 0.01	3.2 × 10^−4^ ± 1 × 10^−4^
GNCA^T235S_T237A_T243S_C69M^	0.05 ± 0.01	3.0 × 10^−4^ ± 1 × 10^−4^
GNCA-All_NN_	0.03 ± 0.01	1.7 × 10^−4^ ± 7 × 10^−5^

The experimental data are provided as a Source Data raw_rates file.

As a control, we also wanted to check if the substitutions that drove GNCA towards being a specific enzyme with an enhanced degradation rate for BZ are due to the ones that are closer to the active site. Therefore, we also studied the specific mutants with substitutions in X, Y, and Z, which lie relatively closer to the catalytic site in β-lactamase enzymes (T235S, T237A, T243S in GNCA^T235S_T237A_T243S^ and T235S, T237A, T243S, C69M in GNCA^T235S_T237A_T243S_C69M^) (see Supplementary Fig. 8). Upon experimental characterization of the activity of these mutants, we observe that these mutations alone have rendered the mutant ineffective to catalyze both BZ and CTX as shown by their turnover rates (Table 1). This emphasizes the importance of allosteric interactions in modulating the function of an enzyme and the key part they play in evolution.

**Fig. 5:**
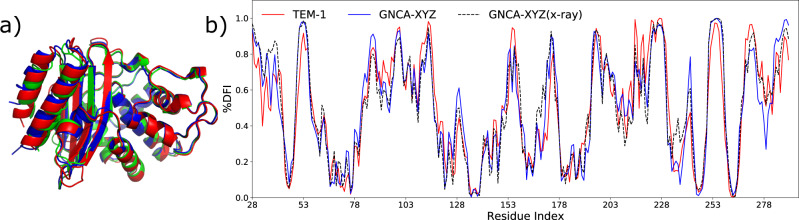
GNCA-XYZ structure obtained through X-ray crystallography reproduces predicted flexibility profile. **a** Superimposing the cartoon representation of the crystal structure of GNCA-XYZ (blue) obtained from X-ray crystallography over the wild-type GNCA (green) and TEM-1 β-lactamase (red) shows that it shares the same 3D fold as the wild-type proteins (RMSD <1 Å). **b** Comparing the DFI profile of the mutant GNCA-XYZ calculated through the dynamics from an MD simulation starting from the crystal structure obtained by X-ray crystallography (black broken line) with the DFI profile of the wild-type TEM-1 β-lactamase (red). We observe that the mutant, as predicted (blue), is able to successfully mimic the rigid regions of TEM-1 β-lactamase, and also its flexible regions with remarkable accuracy. Moreover, the DFI profile of the mutant calculated using the structure obtained through X-ray crystal also matches with our prediction obtained by the mutations performed computationally. This shows the relative robustness of the computational procedure followed in the analysis. The computed data are provided as a Source Data file.

Subsequently, we also obtained the crystal structure of the final engineered enzyme, GNCA-XYZ (see “Methods”). We observed that the substitutions from mutation set X, Y, and Z has preserved the 3D fold of the enzyme (RMSD < 1 Å) (Fig. 5a). We computationally characterized the flexibility profile of the mutant by obtaining its dynamics through an MD simulation using the crystal structure as the starting point and then calculating the DFI flexibility profile (GNCA-XYZ(X-ray)). Afterwards, we compared this flexibility profile with the DFI profile predicted earlier of the mutant and that of wild-type TEM-1 β-lactamase (Fig. 5b). We observe that, as predicted, the flexibility profile of the engineered mutant is very similar to the DFI profile of the wild-type TEM-1 β-lactamase.

### NMR analysis shows dynamic differences between wild-type GNCA and mutant GNCA-XYZ

**Fig. 6:**
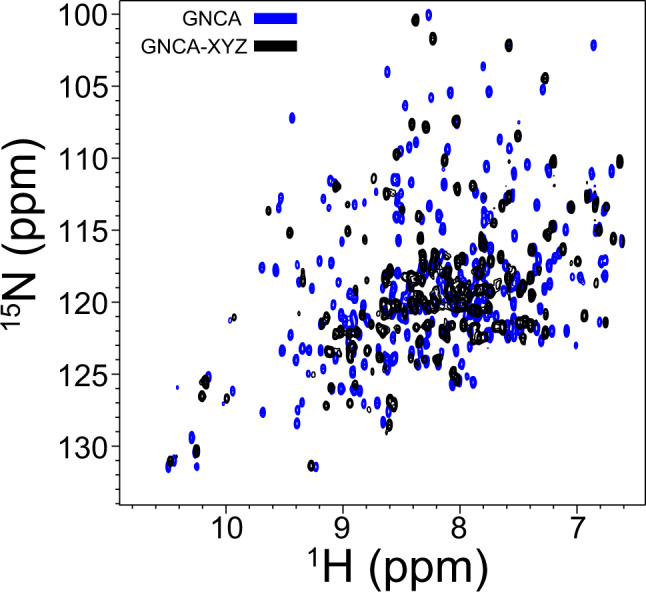
HSQC NMR data indicate that GNCA-XYZ is more dynamic than GNCA β-lactamase. An overlay of GNCA-XYZ (black) and GNCA (blue) ^15^N-HSQC spectra. Both proteins are well-structured as noted in the >3 p.p.m. proton dispersion and consistent with the X-ray structures of a mixed α-helix and β-sheet structure. However, GNCA-XYZ shows spectral features consistent with increased protein dynamics. Specifically, GNCA-XYZ shows fewer resonances, less peak resolution, and increased peak intensity heterogeneity; all are markers of increased protein dynamics relative to GNCA. Data were collected at 850 MHz ^1^H frequency and 30 °C. The experimental data are provided as a Source Data file.

In the previous sections, we have computationally designed GNCA-XYZ function through attempts to modulate its dynamics by substituting hinge positions and regions coupled to hinge positions with the goal of shifting the GNCA dynamic flexibility profile towards that of TEM-1 β-lactamase (Fig. 4). As DFI profiles have been shown to correlate with functional outcomes^27,29^, our hypothesis that the function of the engineered mutant (GNCA-XYZ) should behave more like TEM-1 β-lactamase, with regards to the specificity and catalytic activity. We have shown that the experimental characterization of GNCA-XYZ supports the hypothesis as we observe a significant difference in the turnover rates of GNCA-XYZ with antibiotics CTZ and BZ as compared to ancestral and extant enzymes (Table 1). Moreover, the GNCA-XYZ X-ray structure confirms that the 3D structure is preserved, indicating that the engineered 21 substitutions have introduced changes only in dynamics. In order to further validate the computational predictions that the changes in dynamics govern the function of GNCA-XYZ, we performed solution nuclear magnetic resonance (NMR) spectroscopy experiments. A standard protein NMR experiment couples amide proton and nitrogen atoms giving probes throughout the protein backbone, which results in a fingerprint type identifying 2D spectrum. These HSQC (*h*eteronuclear *s*ingle *q*uantum *c*oherence)-based experiments allow for a qualitative assay of the folded state and structural ensemble of a given protein in solution. One attribute of HSQC data is the proton dimension dispersion, consistent with the X-ray crystallography data, both GNCA and GNCA-XYZ have broad proton dispersion, indicating that both proteins are well-folded in solution. A qualitative assessment of dynamics can also be gleaned by these experiments. For a well-structured rigid protein, there is nearly a one-to-one correlation between the number of NMR spectral resonances and the number of residues, which arises naturally as the HN bond is a *de facto* probe and gives rise to a discreet resonance. In the context of a protein with increased dynamics, the correlation between the resonance number and residue number can diverge away from parity depending on the timescales associated with conformational fluctuations between states. Consistent with a well-folded and relatively rigid protein, the HSQC data from GNCA identifies >90% (253 resonances) of the expected number of resonances, which is consistent with GNCA being rigid. GNCA-XYZ, on the other hand, shows ~60% (158 resonances) of the expected resonances, which is consistent with protein dynamics on the intermediate NMR timescale^64^ (see Fig. 6).

Beyond resonance number and proton dispersion, the HSQC peak resolution and intensity heterogeneity can also serve as qualitative measures of protein dynamics. Comparatively, GNCA-XYZ has lower peak resolution and increased peak intensity heterogeneity, which is consistent with it having increased backbone dynamics over the relatively rigid GNCA protein. Taken together, the NMR data complement the crystallography by showing that GNCA-XYZ is well structured in solution. Similarly, the data support the computational predictions that GNCA-XYZ has increased dynamics compared to GNCA due to the decrease in the total number of hinges after substitutions from the XYZ set as observed from the comparison of the DFI profile of the mutant with the wild-type GNCA β-lactamase (see Fig. 3). This offers validation to the computationally predicted hypothesis of protein dynamics contributing to the regulation of protein function^64^.

## Discussion

In this study, we developed a design principle to evolve the antibiotic activity of ancestral β-lactamase (GNCA) to approach that of its extant counterpart (TEM-1 β-lactamase) by focusing on the differences in their dynamics through utilizing DFI profiles. It has been observed that the two proteins share a relatively high sequence identity (~50%) as well as a common 3D structure^10,13^. Despite these similarities, they are functionally divergent. Specifically, ancestral GNCA β-lactamase exhibits catalytic promiscuity and degrades two types of antibiotics (CTX and BZ) with similar turnover rates. However, the extant TEM-1 β-lactamase is specific with a 10,000-fold increase in turnover rate for CTX and BZ^10,13^.

As shown by our previous studies^9,10,27–29,49,54^, such functional diversity between ancestral and extant enzymes can be explained by focusing on the preserved and evolving features in the flexibility profile of their residues obtained through our computational analysis (DFI). DFI flexibility profiles obtained from MD simulations were used to categorize enzyme residues based on their flexibilities. Thus, allowing us to mimic the evolutionary pathway by manipulating the catalytic activity of the enzyme by subtle shifts in the hinge regions of their DFI profiles. This mechanism of hinge shift was also observed in the evolutionary history of other protein systems like thioredoxin^27^ and GFP^28^. Following this, the hinges in GNCA and TEM-1 β-lactamase were classified based on residue identity and rigidity conservation.

In order to mimic the evolutionary identified hinge shifts, we first focused on a subset of hinges that have altered their dynamics by gaining flexibility. On the other hand, some other flexible sites were rigidified and turned to hinges through substitutions (set X). Through this, we were able to successfully emulate hinge shifts in several regions of the protein (see Fig. 3). Second, we turned our attention to hinges, which have retained their rigidity despite having substitutions. This indicates the critical role played by these residues in the enzyme’s 3D network of interactions to mediate dynamics. However, these common hinges need to be substituted to compensate for the new hinge formations observed by substitution in X set. Thus, we identified Y set substitutions by measuring the long-range dynamic interactions with the X set positions through our DCI metric. Substitutions from set Y, on their own, had a deteriorating impact on the dynamics of the protein (Fig. 3). However, as predicted, together with set X (called set XY), these substitutions compensate for the impact of the previous substitutions and bring the flexibility profile of the mutant very close to the target profile of TEM-1 β-lactamase (Fig. 4).

Last, we fine-tuned the enzyme function by modulating the dynamic allosteric interactions. We focused on the residues with medium flexibility (due to their less-invasive nature upon substitution^29,51,59^), which also exhibit high dynamic coupling with the catalytic sites (DARC spots^9,29,52,59^) obtained through DCI. Of these, a subset was selected that shows stronger coupling with other non-common hinge residues that are not substituted during evolution (set Z). This was a key step in our design strategy as it allowed us to distally modulate the flexibility of these hinge positions without directly mutating them. Adding substitutions in set Z with the previous substitutions (set XYZ), we managed to shift the DFI profile of the engineered mutant towards that of TEM-1 β-lactamase beyond what was accomplished by the XY substitutions alone (Fig. 4). Experimental characterization of the mutant also corroborates with our prediction as we are able to introduce a 10,000-fold disparity in the turnover rates for antibiotics by enhancing its turnover rate catalyzing BZ (by an order of 3) and decreasing the turnover rate for CTX (by an order of 10^4^) (Table 1). These experiments validate the ability to use the hinge-shift mechanism for engineering the desired functional activity.

Overall, we used an approach motivated by conformational dynamics to rationally redesign a promiscuous enzyme, GNCA, toward an enzyme with better efficiency to a specific substrate similar to TEM-1 β-lactamase. First, our dynamics approach uses the hinge-shift mechanism, presented in our earlier studies^9,27,28,55^ highlighting how compensation of enhanced flexibility of rigid (hinge) sites with rigidification of flexible sites modulate the conformational dynamics toward the desired function. Second, our approach uses the protein flexibility profiles as an optimization criterion towards the desired function such that it checks whether the substitutions shift the flexibility profile of the mutant from that of wild type to the flexibility profile of the enzyme with the desired function. The success of our dynamics-based design approach brings to light the importance of protein dynamics and allosteric interactions in engineering new dynamics for an enzyme, which is a current Achilles’ heel in enzyme design^21,22,40,41^. It provides dynamics-based computational design principles to enhance or fine-tune the activity of enzymes based on conformational dynamics and allosteric networks as opposed to the traditional methods to optimize the interaction within a 3D structure near a catalytic site^32,35^.

## Methods

### MD simulation protocol

The dynamics of the wild-type proteins and their mutants were obtained by performing their all atomistic MD simulations. The starting structures for the wild-type proteins were obtained from the protein data bank (PDB) (TEM-1 β-lactamase, from PDB ID: 1BTL^65^ and GNCA from PDB ID: 4B88^13^). The mutants were generated using the mutagenesis tool of PyMol^66^ using the wild-type structures as a template and by replacing the wild-type amino acids with the mutant amino acid templates where the initial rotameric state is selected in order to have minimum steric hindrance in the structure. The software package AMBER17^67^ was used to provide tools for creating the topology files and running the simulations. The topology files are created by loading the starting structures into TLEAP and using the force field ff14SB^68^. The protein hydrogens were added, and the protein was solvated in a 14 Å cubic water box (TIP3P) with neutralizing ions^69,70^. The energy of the system is minimized using SANDER module of Amber17^67^ by minimizing the solvent molecules first and then followed by the whole solution. The water box is then heated up from 0 to 300 K over a duration of 250 ps. The equilibration of density and then a production run is performed by GPU-accelerated PMEMD module of AMBER17^67,71^. During simulations, periodic boundary conditions are used, and bond lengths of all covalent hydrogen bonds are constrained using SHAKE^67^. Direct-sum, non-bonded interactions are cutoff at 9.0 Å, and long-range electrostatics were calculated using the particle mesh Ewald method. During production and heat-up, we use Langevin thermostat to control the temperature at 300 K and Berendsen barostat to adjust the pressure at 1 bar. A time step of 2 fs for the integrator is used for heat-up and production run. All simulations are performed until the dynamic obtained from DFI were converged (see next section).

### Dynamic flexibility index

DFI^9,10,27–29,48–50^ calculates the resilience each residue experiences to force perturbations in the protein. Computation of DFI utilizes the perturbation response scanning (PRS)^48,49,72,73^ technique using small random unit forces as probes to sample the local vibrational ensemble of each residue in the protein. In this model, the protein is coarse-grained using Elastic Network Model (ENM) where each residue is represented by a node at their alpha-Carbon (Cα) atoms, and the bonds between interacting residues are replaced with springs. In this model, the response of the protein in its native state to a perturbative force can be calculated using Linear Response Theory (LRT)^48,72^ as:

1 $${\Delta}{\mathbf{R}}_{3N \times 1} = {\mathbf{H}}_{3N \times 3N}^{ - 1}{\mathbf{F}}_{3N \times 1}$$

where **H** is the *3N* × *3N* Hessian matrix of the protein with *N* interacting residues. It is composed of the second-order derivatives of the harmonic potentials with respect to the components of the position vectors of residues, giving the position covariance of the residue pairs in equilibrium conformation. **F** is the perturbative force vector and Δ**R** is the response vector of residues due to the force. In this model, random unit Brownian kicks are used as perturbative forces in order to mimic the effect of the stochastic nature of forces among water and protein residues in a cell.

Prior work on ENM-based PRS models suggests that such models are able to accurately capture the global dynamics of the protein motion^74,75^. However, as the use of Hessian suggests, this technique is limited by the use of harmonic approximation on a static 3D structure. As a result, the ENM model fails to incorporate the changes in the dynamics of the protein upon changes in the chemistry of the protein by residue substitutions. Therefore, in order to predict the dynamics of the mutants generated in this study, we have performed all-atomic MD simulations on the mutants of GNCA (previous section). The simulations provide us with pairwise correlations in the fluctuations of Cα atoms in the protein in the form of a covariance matrix, which is proportional to Hessian inverse at equilibrium. Thus, Eq. 1 can be rewritten as:

2 $${\Delta}{\mathbf{R}}_{3N \times 1} = {\mathbf{G}}_{3N \times 3N}{\mathbf{F}}_{3N \times 1}$$

where **G** is the covariance matrix obtained from MD simulations, which should be sufficient to gather the differences in dynamics between proteins with a similar native fold by the different chemical composition of the residue. With the use of Eq. 3, the average response profile of the protein can be obtained upon application of random Brownian kicks, uniform in all directions. The displacements of the network are calculated as the random forces, **F** is applied sequentially to each Cα atom in the protein in order to calculate the perturbation response matrix, *A* as,

3 $$A_{N \times N} = \left[ {\begin{array}{*{20}{c}} {\left| {{\mathbf{{\Delta}}}{\mathbf{R}}^1} \right|_1} & \cdots & {\left| {{\mathbf{{\Delta}}}{\mathbf{R}}^N} \right|_1} \\ \vdots & \ddots & \vdots \\ {\left| {{\mathbf{{\Delta}}}{\mathbf{R}}^1} \right|_N} & \cdots & {\left| {{\mathbf{{\Delta}}}{\mathbf{R}}^N} \right|_N} \end{array}} \right]$$

where, $\left| {{\Delta}{\mathrm{R}}^j} \right|_i = \sqrt {\langle\left( {{\Delta}{\mathrm{R}}} \right)^2}\rangle$ is the magnitude of fluctuation response at site “*i*” due to the perturbations at site “*j*” averaged over various random perturbations in all directions. The perturbation matrix provides the net average displacement of the residue from its equilibrium position when all the residues are perturbed by a unit force one at a time. The DFI score of a residue position “*i*” is defined as the ratio of its net response as all the residues in the protein chain are perturbed one by one in a sequential manner and the net displacement of all the residues when everything is perturbed.

5 $${\mathrm{DFI}}_i = \frac{{\mathop {\sum }\nolimits_{j = 1}^N \left| {{\mathbf{{\Delta}}}{\mathbf{R}}^j} \right|_i}}{{\mathop {\sum }\nolimits_{i = 1}^N \mathop {\sum }\nolimits_{j = 1}^N \left| {{\mathbf{{\Delta}}}{\mathbf{R}}^j} \right|_i}}$$

Therefore, a residue with a higher DFI score is more susceptible to random perturbations in the protein and samples the local conformation space more freely, and hence is labeled as a flexible residue. On the other hand, residues with a lower DFI score are more resilient to motions in the protein, and are therefore called rigid residues.

### Convergence protocol for dynamics from MD simulations

As described by Eq. 1, when a Hessian is used to calculate the response of a perturbative force, we are restricting ourselves to a harmonic potential. Therefore, as we sample data from a simulation trajectory in order to calculate the covariance matrix, we are essentially assuming that the data are sampled from a gaussian distribution (because of harmonic potential). In order to achieve appropriate sampling, two of the basic conditions discussed in the sections above have to be met: (i) All conformations sampled must belong to the same distribution. Otherwise, the potential energy well underlying the distribution is different for different configurations in our sample. (ii) The covariance matrix thus obtained should be independent of the choice of the subset of atoms used for fitting coordinates (in order to find the equilibrium coordinates and eliminate global motions). In order to ensure that these two criteria are met following steps are taken.

First, the trajectory is divided into smaller window sizes with different starting points separated by a time lag (25 ns). Three different window sizes (i.e., 25, 50, 75, and 100 ns) are used. For each window size, we calculate a covariance matrix by fitting all the configurations with the first frame of the given window. For fitting, we use only the heavy atoms along the backbone of the protein chain. It should be specified that, for the analysis, the first 100 ns of the trajectory is rejected to avoid relaxation artifacts.

Afterwards, for each window size separately, we obtain the average DFI profile by sampling over each covariance matrix of that window size throughout the trajectory. The average DFI profile for each window size is then compared with others to see if they exhibit common features (namely low flexibility and high flexibility regions). If all the window sizes are sampling from the same potential energy minima, then the final average DFI profiles will be independent of the window size, that is, averaging of DFI profile from 25, 50, and 75 ns window size will give similar results. Therefore, providing us with consistent and converged dynamics. In addition, this will also ensure that the final resultant DFI profile is independent of the choice of a subset of atoms for fitting as the initial coordinates for fitting are different for each window.

If the condition described above is not satisfied, then we consider the trajectory to be not converged and longer simulation times are used. An example of convergence is shown in Supplementary Fig. 9. For all MD runs in the study, the simulation times are provided in Supplementary Table 1 and covariance matrices of length 50 ns are used for the calculations of DFI and DCI profiles.

### Dynamic coupling index

DCI^9,10,28,29,48,51–53^, as the name suggests, is a metric designed to quantify the strength of coupling between residues in a protein network. Similar to DFI (described above), it utilizes the principles like PRS and LRT in order to probe the coupling of a residue “*i*” with another group of residues (*N*_functional_). The DCI score of a residue “*i*” with another residue “*j*” is defined as the ratio of the total displacement at “*i*” when Cα at residue “*j*” is perturbed by a unit Brownian kick to the average displacement of residue “*i*” when all the residues in the protein chain are perturbed by unit force Brownian kicks. It is expressed as:

6 $${\mathrm{DCI}}_i = \frac{{\mathop {\sum }\nolimits_j^{N_{\mathrm{functional}}} \left| {{\mathbf{{\Delta}}}{\mathbf{R}}^j} \right|_i/N_{\mathrm{functional}}}}{{\mathop {\sum }\nolimits_{j = 1}^N \left| {{\mathbf{{\Delta}}}{\mathbf{R}}^j} \right|_i/N}}$$

Here, as earlier, |**∆R**^*j*^|_*i*_ is the response fluctuation profile of residue “*i*” upon perturbation of residue “*j*.” Therefore, a higher DCI score of a residue with the functional sites would imply that perturbation at functionally important sites has a larger impact on that residue as compared to the rest of the protein indicating a higher coupling between the two. On the other hand, a lower DCI score would mean otherwise.

This DCI calculation allows us to compute the dynamic coupling of the positions with the active site. However, we also wanted to calculate pairwise dynamic coupling among NN hinges for set X and between NN and NC hinges for set Y. For that we slightly modify Eq. 6 to compute dynamic coupling index of position “*i*” upon perturbation at position “*j*” as:

7 $${\mathrm{DCI}}_i^j = \frac{{\left| {{\mathbf{{\Delta}}}{\mathbf{R}}^j} \right|_i}}{{\mathop {\sum }\nolimits_{j = 1}^N \left| {{\mathbf{{\Delta}}}{\mathbf{R}}^j} \right|_i/N}}$$

Similar to the earlier criterion, residues with a higher %DCI score (>0.8) will be considered to be highly coupled to residue “*j*.”

### Clustering of proteins based on their DFI profile

In order to cluster the proteins based on their DFI profiles, we compare the percentile ranking of different residues in each protein. We create a data matrix *X*, where the percentile ranked DFI profile of each protein is stored in a column after alignment of their sequence with each other such that for each protein equal number of residues (say “*n*”) will be compared. Therefore, if “*m*” proteins are being clustered, the dimensions of matrix *X* will be *n* × *m*. Afterwards, in an attempt to reduce the statistical noise in the data, we perform dimensionality reduction using singular value decomposition. It is done by firstly reducing the matrix *X* as:

7 $$\left[ X \right]_{n \times m} = \left[ U \right]_{n \times n}\left[ {\Sigma} \right]_{n \times m}\left[ V \right]_{m \times m}$$

where *U* and *V* are the left and right singular vectors of *X*, and Σ is a diagonal matrix with singular values of *X* as its diagonal elements. The singular vectors are orthogonal with respect to each other and represent the orthogonal basis of the vector space of the data. The singular values represent the variance in the data along the corresponding singular vectors. Assuming that the singular values of Σ are arranged in a decreasing order of their magnitude, we select the highest “*r*” singular values such that these contain the largest variance (hence contains the most significant features) in the data. Using these, the data can be reconstructed with lower dimensions as:

8 $$\big[ {X^ \ast } \big]_{m \times r} = \big[ {V^{{\mathrm{T}} \ast }} \big]_{m \times r}\big[ {{\Sigma}^ \ast } \big]_{r \times r}$$

Here, Σ* is the diagonal matrix with “*r*” largest singular values and *V*^T^* is the transpose of the matrix with corresponding “*r*” left singular vectors and X* is the data with reduced dimensions containing “*m*” proteins with “*r*” features ready to cluster. The data in the reduced dimensions, therefore, describe the key differences between the DFI profile of the proteins, such that each row represents the DFI profile of the proteins and the column gives their positions in the principal components. The distance between the proteins can be further visualized in a 2D plot where on each axis we show their respective principal components. Therefore, proteins farther apart in this 2D plot also have larger differences between their DFI profiles. However, proteins closer to each other in the plot also share a larger degree of similarities in their DFI profiles.

In order to cluster, we can compare the proteins based on their features (called principal components). These principal components can be used to show the differences in data by scattering the proteins on a plane with the principal components as their axis as shown in Figs. 3 and 4 (also Supplementary Figs. 5 and 6). Data that lie closer to each other on this plane share more similar features than two data points farther away from each other. Another way of clustering the data is by creating hierarchical trees using a dendrogram. To do so, we calculate the pairwise Euclidean distance between all pairs of proteins (say “*l*” and “*m*”) in the reduced dimensions as:

9 $$d_{lm} = \sqrt {\mathop {\sum }\limits_{i = 1}^r \left( {X_i^l - X_i^m} \right)^2}$$

### Experimental characterization of the mutants

Proteins studied in this work were prepared as we have previously described^76^. Briefly, genes cloned into a pET24 vector with resistance to kanamycin were transformed into *E. coli* BL21(DE3) cells (catalog number: 200131 and vendor: Agilent (https://www.agilent.com/store/en_US/Prod-200131/200131)). Proteins were purified by NTA affinity chromatography, taking advantage of the presence of a His-tag at the C-terminal. Protein solutions were prepared by exhaustive dialysis against 50 mM HEPES buffer and protein concentrations were determined spectrophotometrically using a known value of the extinction coefficient at 280 nm.

Catalytic parameters for the hydrolysis of lactam antibiotics were determined at 25 °C, as we have previously described^13^. Briefly, initial rates were determined from the change in UV absorbance that accompanies substrate hydrolysis and values of the Michaelis constant, turnover number, and catalytic efficiency were calculated by fitting the Michaelis–Menten equation to the profiles of rate versus substrate concentration. In some cases, linear plots were observed, indicating a very large value of the Michaelis constant. In those cases, only the value of the catalytic efficiency was calculated from the experimental profiles.

Denaturation temperatures were determined from differential scanning calorimetry experiments, as we have previously described^13^. Briefly, protein solutions were exhaustively dialyzed and the buffer from the last dialysis step was used as the reference solution in the calorimetric experiments. Equilibration of the instrument was ensured by recording several baselines prior to the experiment with the protein sample. Denaturation temperature values were determined as the temperature corresponding to the maximum of the heat capacity profiles.

### Crystallization, data collection, and structure determination

Crystallization of GNCA-XYZ was done by the counter diffusion technique^77^ using the 24 conditions minimum crystallization screening kit together with the mixPEG at pH 4.0–9.0^77^. Protein solution, at 30 mg/mL in 25 mM HEPES pH 7.0, was loaded in capillaries of 0.2 mm inner diameter, sealed at the top of the capillary, and confronted to the precipitant solutions. After several days, first crystals appeared at the bottom of the capillary, but crystals were let set until data collection. Crystals were extracted from the capillary and cryo-protected by the equilibration with 15% (v/v) glycerol or 20% PEG 200 prepared in the mother liquid prior to flash freezing in liquid nitrogen for transportation and data collection. Crystals were grown in several conditions, but the best diffracting crystals were obtained in the mix of PEG at pH 9.0

Crystals were diffracted at the beamline ID30B of the European Synchrotron Radiation Facility (ESRF, France). Data were indexed and integrated with XDS^78^ and scaled with SCALA^79^ of the CCP4 program suite^80^. Molecular replacement was done using as the search model the coordinates of GNCA, PDB ID. 4B88 in Phaser^81^. Refinement was initiated with phenix.refine^82^ of the PHENIX suite^83^ followed by manual building, water inspection, and ligand identification in Coot^84^. The final refinement was assessed, including Titration-Libration-Screw parameterization. The model was verified with Molprobity^85^ prior deposit at the PDB (ID: 6YRS). Additional details are provided in Supplementary Table 2.

### NMR spectroscopy

The GNCA and GNCA-XYZ genes were incorporated into a pET_24B (+) vector and transformed into BL21 (DE3) *E. coli* cells. Starter cultures were prepared with one colony in 5 mL LB with 38 µg/mL kanamycin and incubated overnight at 37 °C with shaking. The starter culture was used to inoculate 1 L of minimal M9 media (12.8 g Na_2_HPO_4_·7H_2_O, 3.0 g KH_2_PO_4_, 0.5 g NaCl, 1 g ^15^NH_4_Cl, 20 mL of 20% w/v d-glucose, 10 mL 100× MEM vitamin solution, 1 mM MgSO_4_, 100 µM CaCl_2_). Protein expression was induced at 0.6 OD_600 nm_ with 400 µM isopropyl β-d-1-thiogalactopyranoside at 37 °C for 3 h. The resulting cells were harvested at 6000 × *g* for 15 min at 4 °C.

The cell pellet containing overexpressed lactamase was resuspended in 20 mL of lysis buffer (20 mM Na_2_HPO_4_, 500 mM NaCl, pH 7.4) per 1 L of cell pellet, 1 mM phenylmethanesulfonyl fluoride, 5 mM magnesium acetate, 23 µg/mL lysozyme, 2.3 µg/mL DNase, and 2.3 µg/mL RNase. The sample was tumbled at room temperature for 20 min, followed by sonication on ice with S-4000 Ultrasonic Processor (Qsonica) at a 3 s on and 5 s off pulse cycle and 65% power. The resulting lysate was centrifuged at 38,500 × *g* for 20 min at 4 °C. The supernatant was collected using a 0.45 µm filter and loaded onto 5 mL QIAGEN Ni-NTA Superflow column at 1 mL/min rate. The column was equilibrated with 5 column volumes (CVs) of binding buffer (20 mM Na_2_HPO_4_, 500 mM NaCl, 20 mM imidazole, pH 7.4) and washed with 2.5 CVs of 8% (58.4 mM imidazole) of elution buffer (20 mM Na_2_HPO_4_, 500 mM NaCl, 500 mM imidazole, pH 7.4). The lactamase was eluted by a linear gradient of the binding and elution buffers over 9 CVs and ranging from 8 to 70% elution buffer concentration. Fractions were identified by sodium dodecyl sulfate-polyacrylamide gel electrophoresis (SDS-PAGE) and buffer exchanged into NMR buffer (25 mM Na_2_HPO_4_, 250 mM NaCl, pH 6.7) and concentrated to 0.5 mL using 10 kDa cutoff (Millipore Amicon Ultra-4 10 K) for gel filtration chromatography (16XK column with Superdex 200 Prep Grade Resin, GE Healthcare Life Science). SDS-PAGE was used to analyze fractions with high *A*_280_ readings, and selected fractions were combined and concentrated.

The NMR sample was prepared in a 3 mm NMR tube with 2.7% D_2_O, 0.5 mM EDTA, and 550 µM protein concentration in a final volume of 180 µL. All ^1^H-^15^N-HSQC experiments were recorded on a Bruker Avance III HD 850 MHz spectrometer at 303.15 K and equipped with a cryogenically cooled probe. Data were processed in NMRPipe^86^ and analyzed with the CcpNMR Analysis software^87^.

## Supplementary information

Supplementary Information

## Data Availability

Data supporting the findings of this manuscript are available from the corresponding authors upon reasonable request. A reporting summary for this Article is available as a Supplementary information file. Source data are provided with this paper. PDB ID solved: 6YRS.

## Code availability

The code to perform DFI and DCI analysis is available at https://github.com/SBOZKAN/DFI-DCI. Correspondence for the code should be addressed to S.B.O.
