# Supplementary material for: Hinge-shift mechanism as a protein design principle for the evolution of β-lactamases from substrate promiscuity to specificity
Source: Nat Commun. 2021 Mar 25;12:1852. doi: 10.1038/s41467-021-22089-0 (PMC7994827; doi:10.1038/s41467-021-22089-0)
Supplement: Supplementary file 1 — Supplementary Information [file 41467_2021_22089_MOESM1_ESM.pdf]

Hinge-Shift Mechanism as a protein design principle for the evolution of β-lactamase from substrate promiscuity to specificity

Supplementary Information

Tushar Modi[1#], Valeria A. Risso[2,3#], Sergio Martinez-Rodriguez[2]†, Jose A. Gavira[3,4], Mubark D. Mebrat[5,6], Wade D. Van Horn[5,6], Jose M. Sanchez-Ruiz[2,3]*, S. Banu Ozkan[1]*

[1] Department of Physics and Center for Biological Physics, Arizona State University, Tempe, AZ 85287-1504, USA.

[2] Departamento de Quimica Fisica, Facultad de Ciencias, Universidad de Granada, 18071-Granada, Spain.

[3] Unidad de Excelencia de Quimica Aplicada a Biomedicina y Medioambiente (UEQ), Universidad de Granada, 18071 Granada, Spain.

[4] Laboratorio de Estudios Cristalograficos, Instituto Andaluz de Ciencias de la Tierra, CSIC, Universidad de Granada, Avenida de las Palmeras 4, Granada 18100 Armilla, Spain

[5] The Biodesign Institute Virginia G. Piper Center for Personalized Diagnostics, Arizona State University, Tempe, AZ. 85287.

[6] School of Molecular Sciences, Arizona State University, 551 E. University Drive, Tempe, AZ. 85287.

[#] Equally contributed

†Present address: Departamento de Bioquimica, Biologia Molecular III e Inmunologia, Universidad de Granada, Avenida de la Investigacion 11, 18071-Granada, Spain.

*corresponding authors

sanchezr@ugr.es

Banu.ozkan@asu.edu

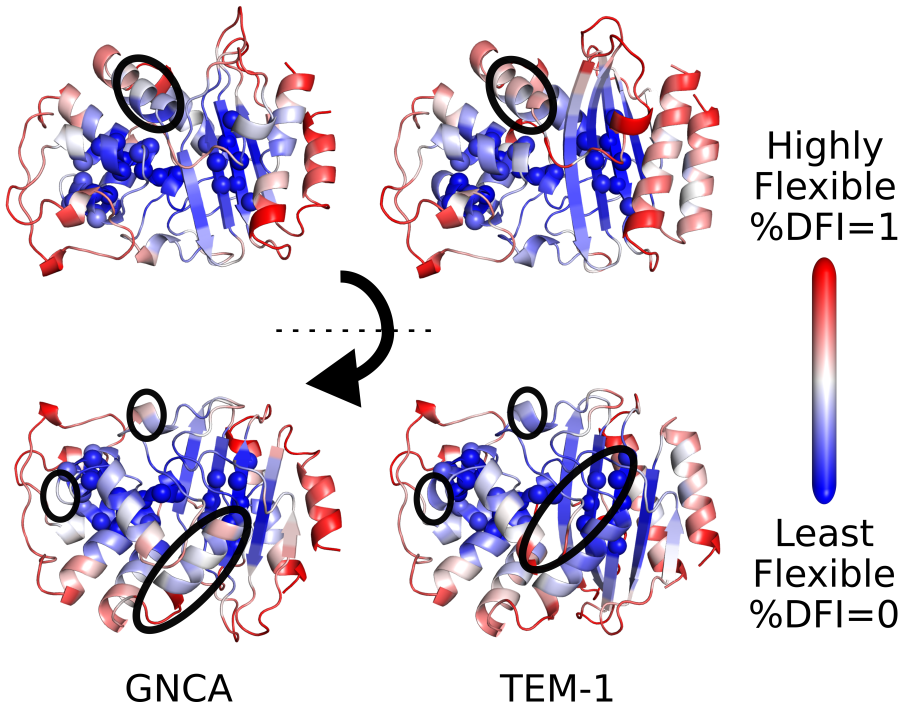

Fig. S1 **Hinge location in GNCA and TEM-1 β-lactamases are shown using %DFI scores in a their color-coded cartoon representations.** The residue positions colored red are highly flexible whereas those colored blue are least flexible, i.e., rigid. The top and bottom panel shows the same protein with different perspective. The residue positions shown as spheres are some of the residues who have retained their rigidity through evolution. On the other hand, we have highlighted residue positions which have undergone hinge-shifts through evolution. We observe a number of residues around the regions with retained rigidity where a shift in their rigidity is observed. This implies a rewiring of the dynamical network of interactions in the protein.

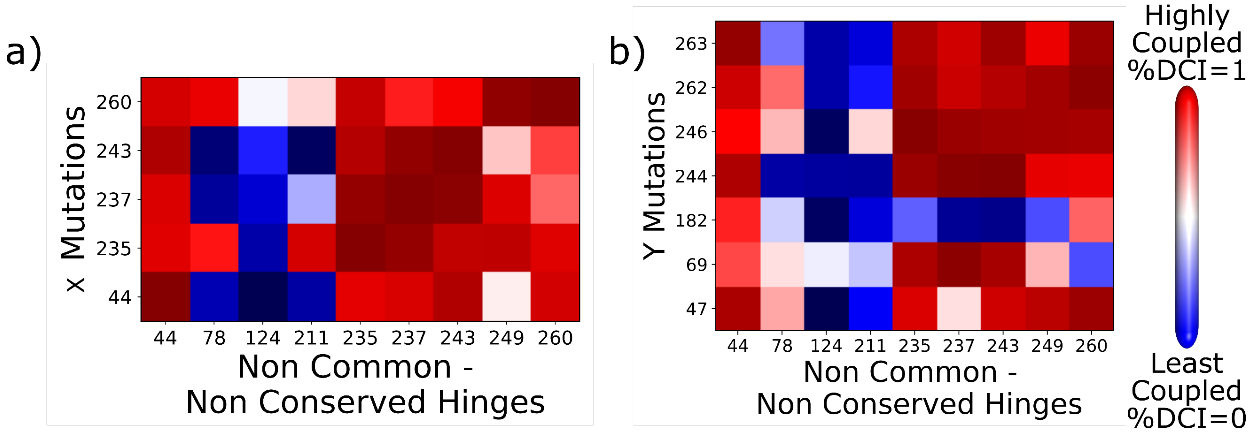

Fig. S2 **Selection criterion for the substitutions in sets X and Y.** The pairwise dynamic coupling of the selected common and sequentially non-conserved hinges comprising (A) X mutation set in GNCA and TEM-1 with other non-common and sequentially non-conserved hinges in GNCA and TEM-1, and (B) Y mutation set in GNCA and TEM-1 with other non-common and sequentially non-conserved hinges in GNCA and TEM-1. The hinge residues selected for the two sets are strongly coupled (%DCI > 0.8) to

other such non-common and non-conserved hinge residues in both GNCA and TEM-1. Calculated data are provided as a Source Data dci_profiles file.

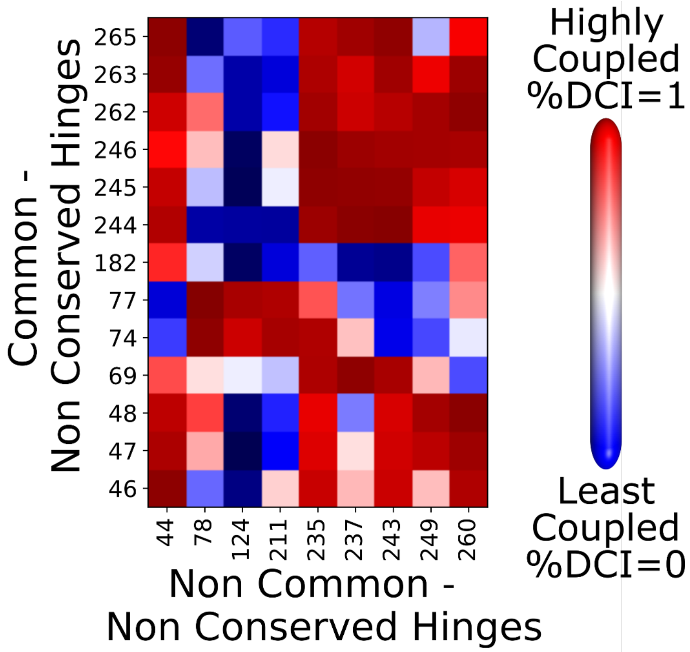

Fig. S3 **Selection criterion for the substitutions in set Y.** The pairwise dynamic coupling of all the common and sequentially non-conserved (NC) hinges comprising with other non-common and sequentially non-conserved (NN) hinges in GNCA β-lactamase is shown. Common and sequentially non-conserved hinges which exhibit a higher coupling (%DCI > 0.8) with other non-common and sequentially non-conserved hinges are selected for substitutions in set Y. For this analysis, we selected such residues from each region in the protein. Therefore, for example, out of residues 262, 263 and 265, only 262 and 263 are selected due to their high coupling with the maximum number of NN hinges; out of 244, 245 and 246, only 244 and 246 are selected, and similarly for others. However, 182 is the only NC hinge in its vicinity which showed higher coupling with few of NN hinges (44 and 260), as a result we included it in the set Y. Calculated data are provided as a Source Data dci_profiles file.

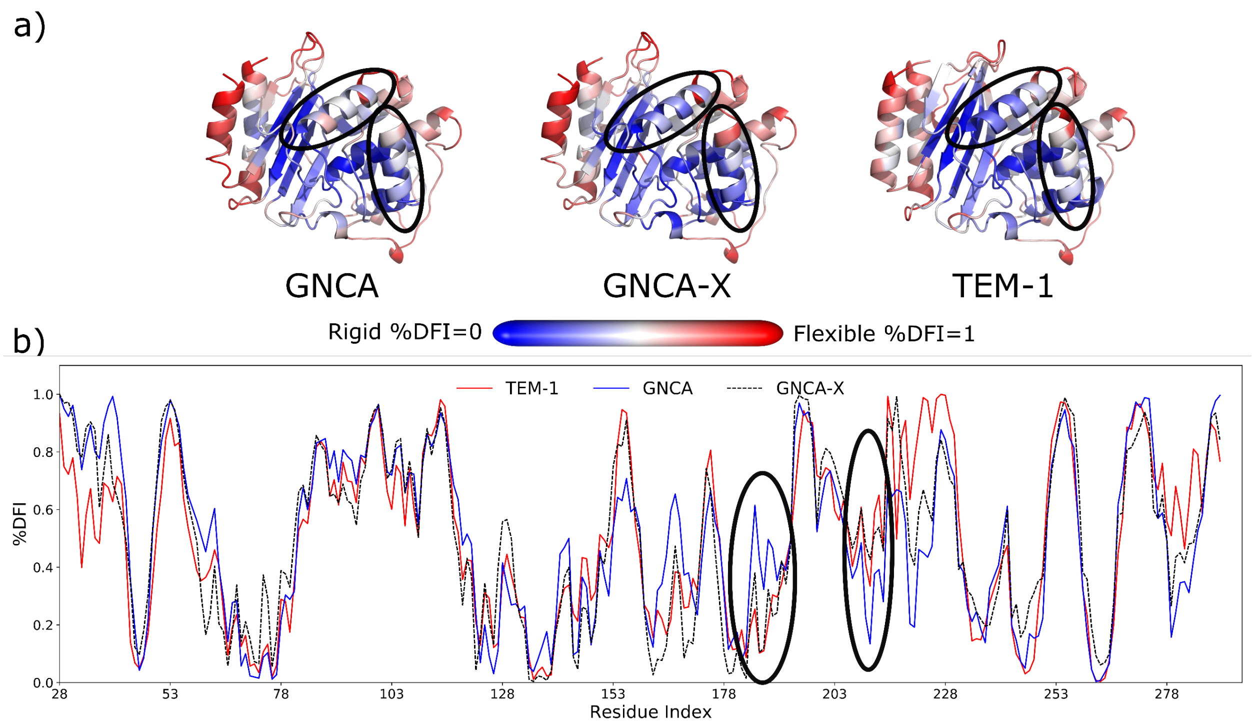

Fig. S4 **The flexibility profile comparison of the wildtype GNCA and TEM-1 β-lactamase with the GNCA mutants. (a)** Color coded %DFI profiles mapped on 3D structures red being flexible and blue being rigid. **(b)** The plot of %DFI values for each position. It is observed that GNCA-Y no longer shares its dynamics with the wildtypes. GNCA-X on the other hand is able to mimic the flexibility profile of TEM-1 particularly around residues 185 and 210 (highlighted) seen in their cartoon representations **(a)**. Calculated data are provided as a Source Data dfi_profiles file.

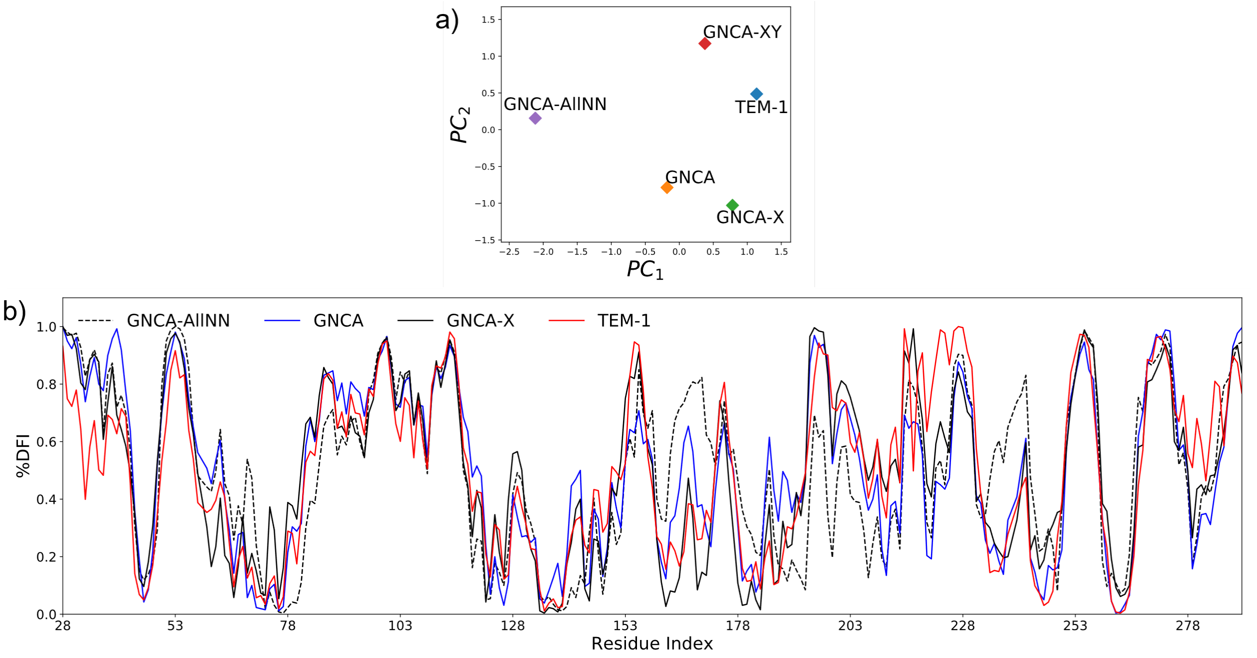

Fig. S5 **The comparison of set X (GNCA-X) that contains a subset of non-common and non-conserved (NN) hinge residues subsitutions with the mutant GNCA-All$_{NN}$ which contains substitutions at all the NN hinges.** We performed MD simulation of the GNCA-All$_{NN}$ after

modelling all mutations at all the NN hinges and then obtained its DFI profile. Comparison of the DFI profile of GNCA-All$_{NN}$ with the that of wildtypes GNCA and TEM-1 β-lactamases is shown through the PCA analysis based on their lowest two principle components **(a)** and through the differences in in their flexibility profiles per residue positions, shown as a plot **(b)**. We observe that mutating all the NN hinges significantly alter the dynamics of the GNCA mutant. Particularly comparing the DFI profiles of each position reveals that mutating all of the NN hinges (broken black line) impacts dynamics of the regions around the catalytic site 70, 166 and 234, suggesting that these substitutions could be damaging to the function. On the other hand, the impact of the mutation from set X, which contains only a select number of non-common and non-common hinges, on dynamics in these regions also impact the dynamics but not so severe (black solid line). This analysis also suggests that substitution of NN sites alone is not enough to modulate function. Calculated data are provided as a Source Data dfi_profiles file.

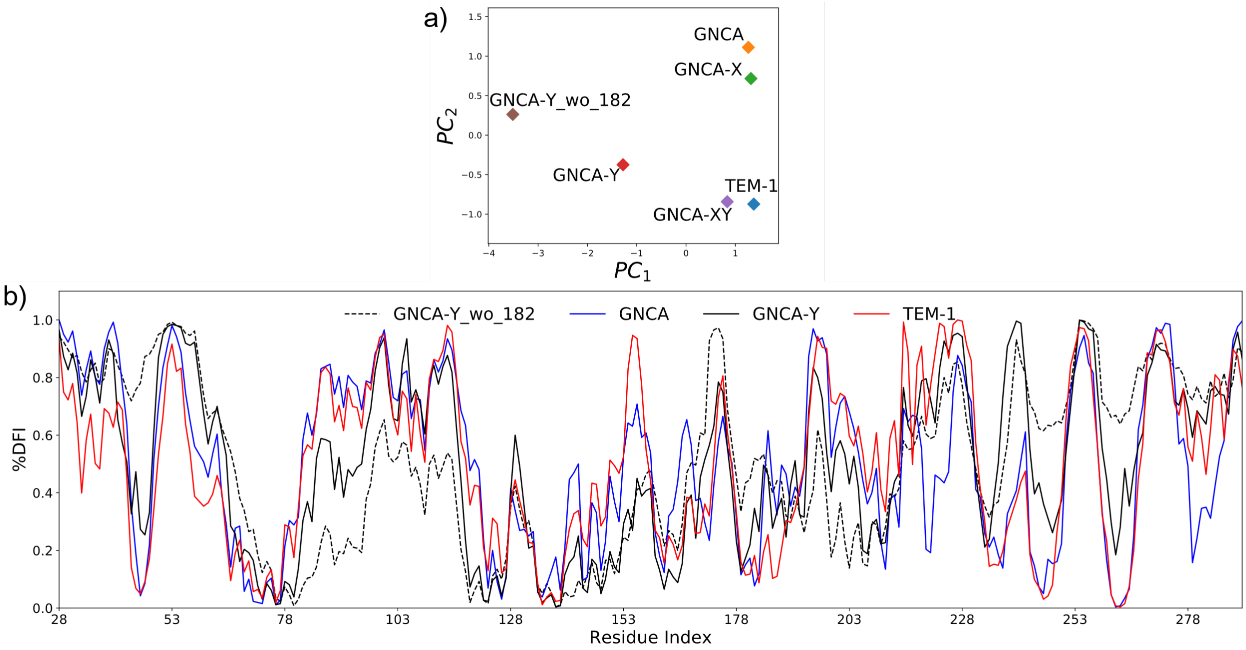

Fig. S6 **The effect of mutations from set Y with (GNCA-Y) and without the mutation T182M (GNCA-Y_wo_182)**. **It** can be observed by clustering their DFI profiles with the wildtypes GNCA and TEM-1 β-lactamases **(a)** and also by comparing the differences in their DFI profiles per position (B). From (A), we observe that the mutations from set Y without the T182M mutation shifts the dynamics of the GNCA mutant farther away from those of wildtypes. This also provides a strong evidence about the importance of T182M mutation in its dynamics. This can also be observed from **(b)** where we observe that the variant (broken black line) exhibits a very different dynamics particularly in several regions 78-110, around residue 200 and 240-260 when T182M mutations is not present. On the other hand, the impact of the mutation set Y on dynamics in these regions is not so severe when we incorporate the T182M mutation (black solid line). Calculated data are provided as a Source Data dfi_profiles file.

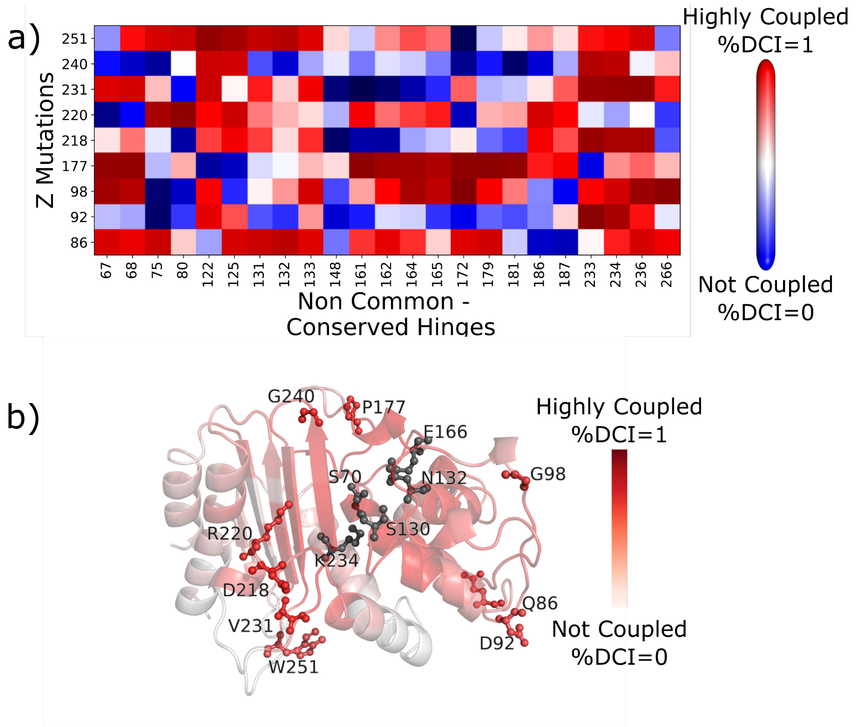

Fig. S7 **Selection criterion for the substitutions in set Z. (a)** The coupling of residues selected for substitutions in set Z with the non-common and sequentially non-conserved hinges. The positions that exhibit higher coupling (%DCI > 0.8) with the non-common and sequentially non-conserved hinges are selected for mutations. Apart from this, these residues also have a medium flexibility (0.3 < %DFI < 0.5) and are distally located from the active site (> 8 $\AA$). **(b)** In addition, the residues in set Z are also coupled to the active site as shown by the cartoon representation of GNCA β-lactamase where each residue is color coded based on corresponding dynamic coupling with the active site (%DCI score). Red colored residues are the highest coupled (%DCI=1) and white are the least coupled (%DCI = 0). The residues in set Z are shown as sticks. We can observe that these also exhibit high dynamic coupling with the active site (%DCI > 0.8). Calculated data are provided as a Source Data dci_profiles and dfi_profiles file.

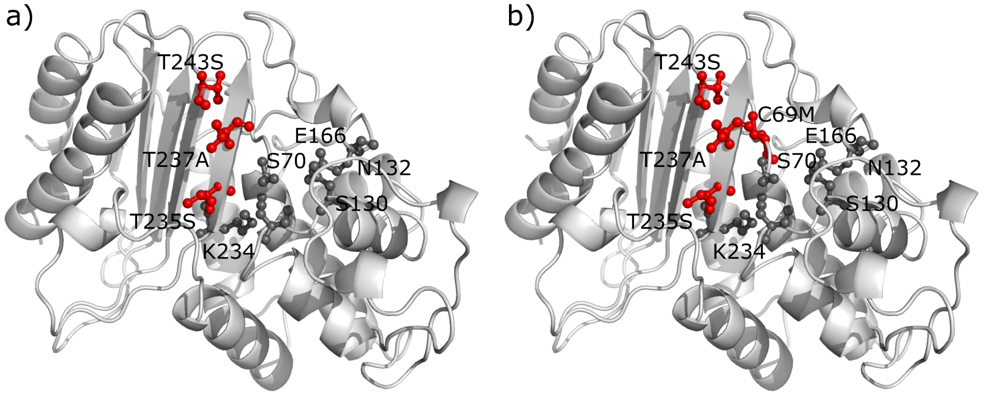

Fig. S8 **Substitutions closer to the catalytic site in GNCA from Set X, Y and Z**. These were performed in **(a)**: T243S, T237A, T235S; and **(b)**: T243S, T237A, T235S, C69M. We observe that upon

performing mutations only closer to the catalytic site were derogatory for the function as both of them have made mutants ineffective for degradation of both the antibiotic benzyl-penicillin and cefotaxime (see Table 1).

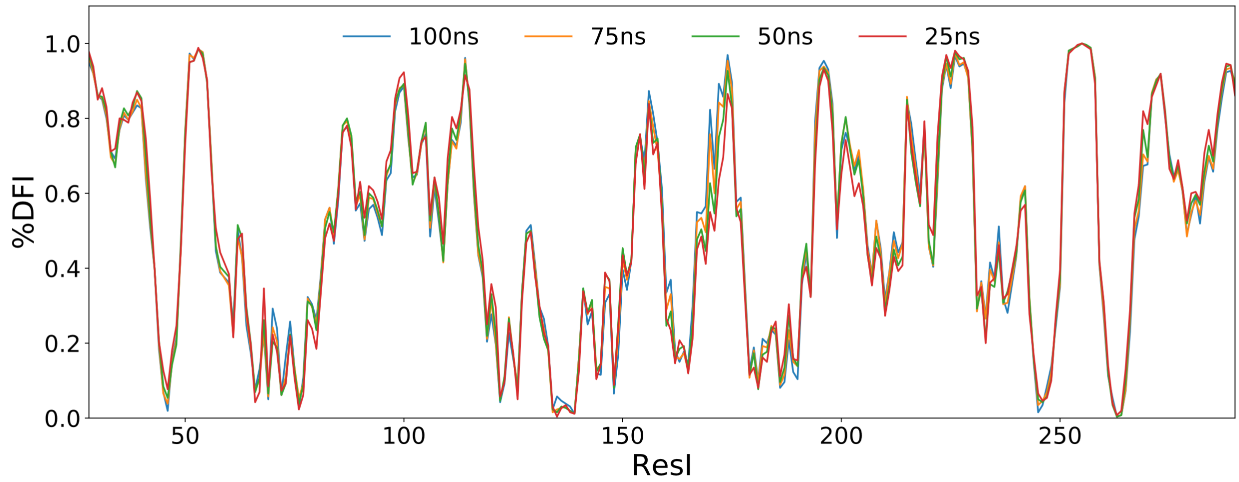

Fig. S9 **Comparison of the percentile ranking of the average DFI profile obtained using covariance matrices calculated from time windows of different sizes (25ns, 50ns, 75ns and 100ns).** These time windows are obtained by sampling the trajectory through a moving window which is shifted by 25ns. If the %DFI profile obtained from different window sizes give a consensus result for the low flexibility region as well as a very low discrepancy of high flexibility region, then the covariance matrices are considered to be sampling from the converged dynamics of a single well. Here we have shown an example of %DFI profiles sampled from different windows from a 1000ns simulation of GNCA-XYZ. The observed high consensus between their DFI profiles give us the confidence that simulation is converged, and the obtained DFI profile reflects the flexibility profile from a well equilibrated single energy well.

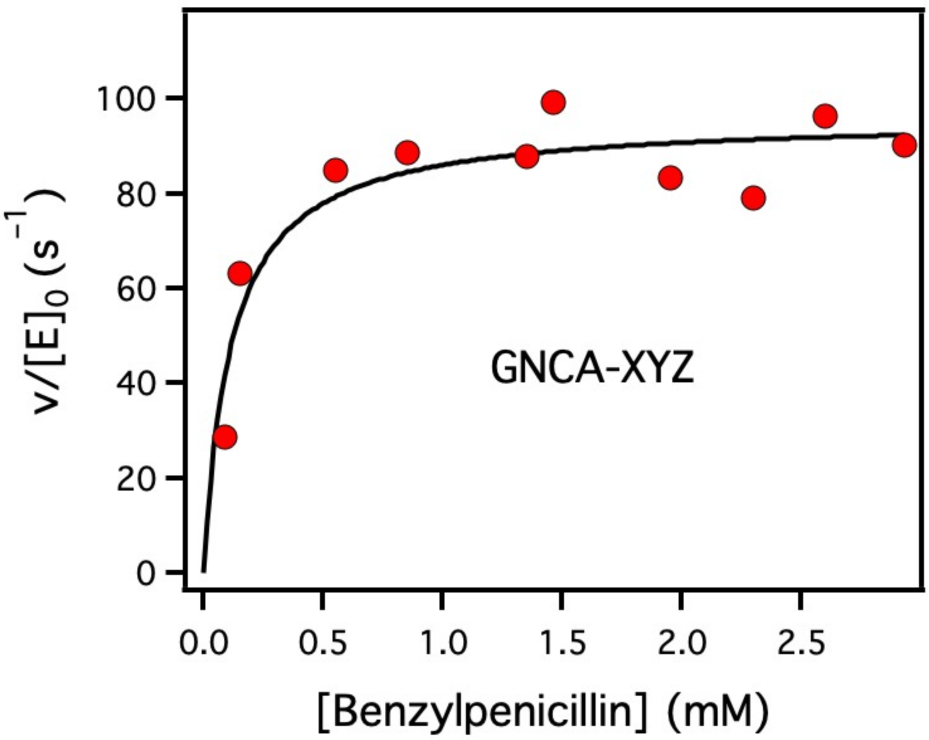

Fig. S10 **Catalysis of in vitro hydrolysis of Benzylpenicillin by ancestral β-lactamase GNCA-XYZ.** Representative plot of initial rate versus substrate concentration. The continuous lines are the best fits of the Michaelis-Menten equation. The values obtained for the Michaelis-Menten parameters are collected in Table 1. Calculated data are provided as a Source Data raw_rates file.

Table S1: The Length of molecular dynamic simulation for each protein.

**Protein**	**Length (ns)**
TEM-1	400
GNCA	400
GNCA-X	400
GNCA-Y	400
GNCA-XY	600
GNCA-XYZ	600
$GNCA^{T235S\_T237A\_T243S}$	400
$GNCA^{T235S\_T237A\_T243S\_C69M}$	400
$GNCA\text{-}All_{NN}$	1000
GNCA-Y_wo_182	400

Table S2. Data collection and refinement statistics. Statistics for the highest-resolution shell are shown in parentheses. Calculated data are provided as a Source Data PDB Validation Report.pdf file.

Source	ESRF ID30B
PDB ID (PDB validation report in supplementary data)	6YRS
Resolution range (Å)	48.73 - 1.7 (1.76 - 1.7)
Space group	P 1 21 1

a, *b*, *c* (Å)	47.35, 81.40, 61.04
(°)	94.58
No. unique reflections	49578 (4871)
Multiplicity	3.0 (2.9)
Completeness (%)	97.68 (96.72)
Mean I/sigma(I)	12.03 (1.30)
Wilson *B* factor ($Å^2$)	29.69
R-merge (%)	4.46 (77.17)
CC1/2 (%)	99.8 (58.8)
Reflections in working / test sets	49571 / 2531
R-work (%)	17.60 (30.96)
R-free (%)	20.85 (33.13)
Atoms (non-H)	4441
macromolecules	4049
ligands	136
solvent	256
Protein residues	504
RMS (bonds) (Å)	0.012
RMS (angles) (°)	1.11
Ramachandran (%)	
favored	98.19
allowed	1.81
Average *B* factor ($Å^2$)	41.92
biomolecules	40.78
ligands	66.09
solvent	47.08
